# Mining the sustainability of takeaway businesses in online food delivery service supply chain

**DOI:** 10.1016/j.heliyon.2024.e27938

**Published:** 2024-03-10

**Authors:** Longxiao Li, Zusheng Zhang, Xixi Li, Jiafu Su, Yanling Jiang, Jun Cao, Fangsu Zhao

**Affiliations:** a School of Business Administration, Chongqing University of Science and Technology, No. 20, East University Town Road, Shapingba District, Chongqing, 401331, China; b College of Mechanical and Vehicle Engineering, Chongqing University, No.174 Shazhengjie, Shapingba District, Chongqing, 400044, China; c Teaching Affairs Department, Chongqing Energy College, Chongqing, 402260, China; d International College, Krirk University, Bangkok, 10220, Thailand

**Keywords:** Sustainability mining, Triple bottom line, Takeaway businesses' sustainability, Online food delivery, Service supply chain, Bayesian best-worst method, HDBSCAN

## Abstract

The online food delivery service supply chain constitutes a crucial element in achieving sustainable development goals. With its prosperity, an increasing number of takeaway businesses are drawn to this sector. As their numbers rise, issues such as profitability resilience, environmental friendliness, and fulfillment of social responsibility emerge, posing potential disruptions to the service supply chain. Against this backdrop, our endeavor is to mine the sustainability of takeaway businesses using the triple bottom line. We propose a two-stage approach involving the Bayesian best-worst method and a data mining technique to derive the weights of sustainability criteria and the clusters of takeaway businesses. Subsequently, a case study is conducted focusing on takeaway businesses on the *Ele.me* platform in China. The results highlight economic sustainability as the most valued criterion, followed by social and environmental sustainability. Clustering outcomes reveal four distinct levels of sustainability, with a stronger performance in social sustainability compared to environmental and economic dimensions. Further discussions explore the relationship between sustainability levels, cuisine categories, and business size. Consequently, this study suggests an effective approach for advancing sustainability initiatives within the online food delivery service supply chain.

## 1 Introduction

Facilitated by online food delivery (OFD) platforms such as *Deliveroo*, *Uber Eats*, *DoorDash*, *GrubHub*, *Meituan*, and *Ele.me*, an escalating number of conventional restaurant businesses have seamlessly integrated into the OFD service supply chain, encompassing takeaway businesses, delivery personnel, and end consumers. However, as the influx of takeaway businesses into this supply chain continues to grow, concerns regarding economic, environmental, and social sustainability have surfaced, posing a potential threat to the overall integrity of the service supply chain.

The sustainability of takeaway businesses is of paramount significance [1], given that their inability to maintain sustainable practices could result in detrimental consequences for key stakeholders such as the OFD platform, end consumers, and delivery personnel across economic, environmental, and social dimensions. For instance, a decrease in the number of active businesses on the OFD platform may impede the efficient promotion of environmentally friendly utensils, thereby compromising the environmental sustainability of both the takeaway businesses themselves and the platform [2]. Furthermore, a reduction in active businesses could diminish the profitability of the OFD platform, adversely impacting end consumers by limiting their access to the convenience of online ordering. Simultaneously, delivery personnel may face reduced earnings and potential unemployment due to a decline in delivery orders.

Recognizing the pivotal role of takeaway enterprises within the OFD service supply chain and the adverse effects of their unsustainable development across economic, environmental, and social sustainability dimensions, our objective is to assess the extent of sustainable development among takeaway businesses. Given the increasing prominence of sustainability as a business strategy [[3], [4], [5]], we integrate the three dimensions of sustainable development within the takeaway sector with Elkington's Triple Bottom Line (TBL) framework. The TBL framework encompasses pillars of environmental, economic, and social sustainability [6], emphasizing the need for a comprehensive and balanced approach to ensure the long-term viability of the OFD service supply chain.

In the realm of environmental sustainability, the rise of OFD services has transformed eating habits, enabling individuals to enjoy a diverse range of cuisines from home [7]. However, this convenience has fostered a growing preference for disposable packaging, leading to a significant increase in single-use products and the generation of substantial non-degradable waste. For instance, China annually produces approximately 30 million tons of packaging waste, with only about 20% being recycled [2]. Particularly in China, the top 10 cities in waste production collectively contribute 30% of the country's takeaway packaging waste, with eastern cities exhibiting the highest per capita waste generation [8]. Amid escalating concerns regarding packaging waste, in 2020, the National Development and Reform Commission (NDRC) and the Ministry of Ecology and Environment jointly issued directives aimed at reinforcing efforts to address plastic pollution within the OFD industry for the first time [9].

In terms of economic sustainability within the OFD service supply chain, the influx of small-scale restaurant businesses into online operations has led to significant revenue growth through increased order volume and frequency [10], without the need for expanding seating capacity or front desk staff [11]. However, heightened market competition and a higher rate of business openings have rendered businesses more vulnerable [12]. Data from the *Ele.me* OFD platform shows that a significant portion of takeaway businesses remains inactive with subpar quality. Moreover, the typical commission percentage charged by OFD platforms [13] forces businesses to increase prices, ultimately shifting the burden to customers, thus posing a risk of disruption to the OFD service supply chain. In response, *Meituan*, a Chinese OFD platform giant, has reduced commissions for takeaway businesses, including capping fees at 5% in 2021 [14], to ensure the continued presence of these businesses. Similarly, in 2022, the 10.13039/501100010453NDRC introduced policies advocating for further reductions in commission fees for takeaway businesses by OFD platforms, aiming to lower operational costs [15].

Beyond environmental and economic sustainability, social sustainability is increasingly vital in the OFD service supply chain. Food consumption fosters social engagement among stakeholders [16]. Uber Eats, for instance, employs over 100,000 delivery personnel as of March 2021, offering significant job opportunities [17]. However, overlooking social issues like food safety poses risks to consumer satisfaction and business reputation [18]. In 2023, the State Administration for Market Regulation and Ministry of Commerce emphasized the need to strengthen the accountability of food delivery entities for food safety, fulfill obligations under food safety laws and regulations, and promote the widespread use of food safety seals [19]. The expansion of OFD services intensifies social sustainability challenges [20], including inadequate social security coverage [21,22] and increased traffic violations [23] among OFD riders. These issues contribute to rising job insecurity and financial instability [24]. Takeaway businesses are thus recognizing the importance of promoting social sustainability through initiatives like ensuring food safety and enhancing well-being [16].

In the rapidly evolving landscape of sustainable development, the role of takeaway businesses stands out as a potential linchpin in the pursuit of Sustainable Development Goals (SDGs) [16]. However, despite their evident significance, a notable void exists within the Chinese restaurant industry concerning the application of sustainability criteria. Moreover, within the intricate web of the OFD service supply chain, studies specifically targeting the sustainability of takeaway businesses remain conspicuously scarce [25]. This gap not only reflects a deficiency in current research but also underscores a critical need for comprehensive frameworks that can effectively evaluate the sustainability of these businesses. Thus, the objective of this study is to develop a comprehensive framework for evaluating the sustainability of takeaway businesses, integrating multiple TBL criteria. We employ a multi-criteria decision-making (MCDM) approach, specifically utilizing the Bayesian best-worst method (BWM) [26].

Leveraging the identified sustainability criteria and aggregated values, we employ data mining techniques to discern the sustainability levels of takeaway businesses. As the number of businesses experiences a significant surge, the data volume and dimensionality also escalate, underscoring the critical need for OFD platforms to extract pertinent information for informed decision-making. In China, for instance, the count of takeaway businesses on OFD platforms exceeded 3.6 million as of mid-2020 [27]. In this scenario, the clustering method emerges as a promising solution to unveil the characteristics and structure embedded in vast datasets [28]. On this basis, we employ the Hierarchical Density-Based Spatial Clustering of Applications with Noise (HDBSCAN) method to cluster takeaway businesses based on sustainability criteria. This method allows for a nuanced understanding of the diverse sustainability levels within the takeaway business landscape, contributing valuable insights to enhance the effectiveness of sustainability initiatives in the OFD service supply chain. To exemplify the two-stage approach in mining the sustainability of takeaway businesses, we conduct a case study involving businesses and marketing managers from the *Ele.me* OFD platform in China.

The remainder of the paper is organized as follows. Section 2 reviews the sustainability in the restaurant industry. Section 3 describes the two-stage approach comprising the Bayesian BWM and HDBSCAN. Section 4 conducts a real-world case study of takeaway businesses in China. Section 5 further discusses the obtained results of the case study. Conclusions and future research directions are given in Section 6.

## 2 Related work

As a fusion of the restaurant industry and instant delivery, the dietary practice of takeaway indeed adds convenience to people's lives, but it also comes with potential adverse impacts. Issues within takeaway businesses, such as profitability resilience, environmental considerations, and the fulfillment of social responsibilities, may result in the disruption of the OFD service supply chain. To address these concerns, takeaway businesses are urged to make changes. Consequently, we strive to establish a sustainability evaluation framework for takeaway businesses based on the TBL. To achieve this goal, we review relevant literature on the topic of sustainability in the restaurant industry.

### 2.1 Environmental sustainability

#### 2.1.1 Certifications and initiatives for sustainability

In the realm of sustainable development, greater attention is directed toward ecological imperatives. The initial stride for restaurant businesses to attain sustainable development involves engaging in sustainable development projects or acquiring pertinent certifications from organizations. These certifications encompass initiatives like Green Restaurant, the Energy Star Program, TFC Recycling, ConSERVE Solutions for Sustainability, Sustainable Foodservice, and Green Seal [18,29]. In the meantime, some restaurants are increasingly focusing on providing sustainable food options and menus for their customers [[30], [31], [32]], a move that reflects their dedication to sustainable practices. This involves supporting local and seasonal products, as well as incorporating organic, ecological, and healthy items, while also transparently listing food ingredients on their menus.

#### 2.1.2 Energy and resource conservation

For restaurants, the preparation and production of food involve the consumption of energy. To reduce resource and energy use and greenhouse gas emissions, various sustainable actions are proposed, including energy audit programs, adopting energy-saving equipment, and low-energy consumption design techniques [[33], [34], [35]]. After the preparation phase is completed, the food delivery has led to a surge in the number of delivery riders, resulting in a significant increase in motor vehicle-based food delivery trips. While most riders opt for scooters or motorcycles, which are plug-in electric vehicles [36], their extensive usage contributes to heightened CO2 emissions [37,38]. Additionally, e-bikes, a primary mode of transportation for food delivery, contribute to environmental concerns due to the disposal of lead-acid batteries [39]. Consequently, the convenience of food delivery services poses a threat to urban environmental sustainability.

#### 2.1.3 Packaging and waste management

When individuals opt for takeaway over dining in, there is a substantial increase in the consumption of food packaging, contributing to environmental issues as one of the influencing factors. Therefore, it is essential to mitigate the waste of packaging materials in food service operations by reducing excessive or unnecessary food packaging [[40], [41], [42]]. In addition to minimizing over packaging, selecting environmentally friendly and biodegradable packaging or tableware for food containment emerges as a premium alternative [25,41,43], aligning with the growing demand for sustainability. Additionally, the utilization of disposable tableware is often deemed necessary for maintaining hygiene and safety standards in the context of OFD. However, there exists a pressing need for concerted efforts to minimize the reliance on disposable items, such as plastic spoons, bowls, and chopsticks. Whenever feasible, a shift towards the adoption of recyclable paper products is advocated [33,43]. Furthermore, the pervasive issue of microplastic pollution resulting from the widespread use of disposable tableware and small packaging is silently undermining public health and disrupting ecological integrity on a global scale [8,44,45]. In the post-consumption phase, food waste also emerges as a significant concern. Implementing food recycling and donation programs, alongside initiating food waste audits and tracking [33,46,47], becomes crucial in reducing the overall amount of wasted food. Therefore, effective mitigation strategies, including rational waste classification and policy enforcement, are imperative to safeguard environmental sustainability [44].

### 2.2 Economic sustainability

#### 2.2.1 Direct economic sustainability

In the realm of the TBL, economic sustainability holds paramount importance, particularly for businesses in the catering industry. Achieving economic sustainability for them involves considerations across several key facets. Direct profit is the most immediate indicator, primarily demonstrated by effectively managing losses and surpluses in food service operations to maximize attainable profits [10,18,25,34]. Generating market opportunities, by placing significant emphasis on the market and cultivating marketing advantages, serves as a profitable avenue for local restaurant businesses, supply chain partners, and other relevant entities [34]. Similarly, achieving profitability can be realized through streamlining the procurement process. This involves reducing the complexity of the ordering process and adopting single-source purchasing, thereby saving both staff time and money [41]. Moreover, at a macro level, the increasing presence of takeaway businesses on OFD platforms supports the survival of physical restaurants during the COVID-19 pandemic and ensures the supply of essential goods during crises [20]. Additionally, they reshape consumer consumption patterns [48] and accelerate the integration of online and offline consumption [49], significantly contributing to the OFD industry and nighttime economic growth [8].

#### 2.2.2 Indirect economic sustainability

In the realm of OFD, sustainable profitability often hinges on the speed of delivery. Providing customers with a faster and more convenient OFD service not only reduces customer waiting time but also enhances patronage, thereby creating a distinct economic advantage [10,11]. Incorporating an e-commerce element, the indirect sustainability of profitability in OFD is achieved through fast refund processes. Providing fast refund or exchange services enhances customer satisfaction, increases purchase frequency, and ultimately boosts customer loyalty, contributing to heightened sales and overall profitability for the business [50,51]. This aligns with the reality that some takeaway businesses on the OFD platform now offer expedited refund options.

### 2.3 Social sustainability

In addition to environmental and economic sustainability, the significance of social sustainability for takeaway businesses cannot be overstated. This is notably evident in areas such as food safety, employment and social welfare, and community prosperity.

#### 2.3.1 Food safety and quality

First and foremost, ensuring the provision of safe food to customers is an essential baseline for takeaway businesses, particularly for those actively embracing green development practices [18,34,41]. After fulfilling the fundamental foundation of food safety, takeaway businesses should strive to offer customers exceptionally delicious cuisine. This is crucial because, in the OFD environment, factors such as the freshness of ingredients, the temperature of the food, and the quality of packaging become pivotal elements influencing customers’ decision to revisit [11,52]. By prioritizing customer satisfaction, a restaurant can progressively establish a positive business image within the local community, positioning itself as a model for others to emulate. This is especially critical for takeaway businesses, as shaping a favorable business image is crucial [32,33].

#### 2.3.2 Community engagement

While providing customers with OFD services, takeaway businesses simultaneously contribute to member connections, community engagement, and overall prosperity through the establishment of a community-embedded business, driven by job creation, innovative culinary offerings, and other activities [31,34,35], such as OFD riders. Besides creating job opportunities, the proliferation of OFD services exacerbates social sustainability challenges, encompassing deficiencies in social security coverage [21,22] and heightened occurrences of traffic violations among OFD riders [23]. Consequently, takeaway businesses and OFD platforms are increasingly acknowledging the significance of fostering social sustainability by implementing initiatives aimed at enhancing community engagement.

#### 2.3.3 Employee training and sustainable practices

For the attainment of social sustainability, ongoing training for employees in takeaway businesses is indispensable. This involves providing them with vocational training on sustainability initiatives, including areas such as energy conservation and the promotion of sustainable, healthy food practices [18,35,41]. Besides, actively participating in local procurement by responsibly sourcing locally grown and seasonal products serves a dual purpose—supporting the local economy and potentially realizing cost savings while mitigating environmental impact [41,47].

Extensive research has laid the theoretical foundation for our study, primarily concentrating on the environmental and resource aspects of restaurant operations. However, limited attention has been given to the sustainability of takeaway businesses within the OFD service supply chain. Our focus is on the environmental, economic, and social sustainability impacts arising from post-production packaging and delivery issues in the takeaway context. These factors encompass disposable tableware use, instant delivery, OFD personnel, food safety, and online reviews. In response, we propose an MCDM framework for sustainability evaluation in takeaway businesses and subsequently conduct a clustering analysis to identify their sustainability levels.

## 3 A two-stage approach comprising the bayesian BWM and HDBSCAN

In this section, we propose a two-stage approach to sustainability evaluation and subsequent cluster analysis.

In the first stage, we use a Bayesian BWM to determine the weights of sustainability criteria. Given that the chosen method involves selecting appropriate evaluation criteria from alternatives and comparing the importance of identified criteria, this study utilizes a questionnaire survey method to harness the insights of both the academic and industry regarding the sustainability evaluation of takeaway businesses. The study was approved by the Academic Ethics Committee of Chongqing University of Science and Technology (reference number 2023LLSC0237). All participants were informed about their role in answering the questionnaires, and all participants provided informed consent to participate.

Then, we employ the HDBSCAN method to perform a clustering analysis of data points under sustainability criteria.

### 3.1 Bayesian best worst method

Considering multiple attributes within the TBL, we utilize a Multi-Criteria Decision-Making (MCDM) method to allocate appropriate weights to the criteria and compute aggregated scores. This paper employs the Best-Worst Method (BWM) [53] due to its appealing features: The structure of BWM provides more reliable pairwise comparisons while mitigating possible anchoring bias, and it is one of the most data (and time) efficient weighting methods, which could also provide a consistency check [54]. The method has been applied to solve problems in many scenarios, such as sustainable product-package design [55], logistics distribution channel selection [56], logistics performance measurement [57], and many more, see Ref. [58].

The BWM has shown its applicability for the MCDM problem, and several extended versions have also been developed [26,53,59,60]. Of these, we adopt the Bayesian BWM. Given that decision-makers (DMs) are a pool of people, the Bayesian BWM provides an ideal way to determine the aggregate weight from a probabilistic perspective [26]. Specifically, it involves the following steps:

*Step 1* Determine a set of decision criteria $C=\left\{c_{1},c_{2},\ldots ,c_{n}\right\}$.
*Step 2* The decision-maker (DM) k selects the best $\left(c_{B}\right)$ and the worst $\left(c_{W}\right)$ criteria from C. In this step, the DMs are not required to perform pairwise comparisons, they only identify the best or most important criterion and the worst or least important criterion.
*Step 3* The DM k performs the pairwise comparison between the best criterion and the other criteria using a number between 1 and 9. The resulting Best-to-Others vector is $A_{B}^{k}=\left(a_{B1}^{k},a_{B2}^{k},\ldots ,a_{\text{Bn}}^{k}\right)$, k=1,…,K, where $a_{\text{Bj}}^{k}$ denotes the preference for the best criterion $c_{B}^{k}$ over other criteria $c_{j}\in C$ for DM k.
*Step 4* The DM k performs the pairwise comparison between the other criteria and the worst criterion using a number between 1 and 9. The resulting Others-to-Worst vector is $A_{W}^{k}={\left(a_{1W}^{k},a_{2W}^{k},\ldots ,a_{\text{nW}}^{k}\right)}^{T}$, k=1,…,K, where $a_{\text{jW}}^{k}$ denotes the preference of the criterion $c_{j}\in C$ over the worst criterion $c_{W}^{k}$ for DM k.
*Step 5* Estimate the probability distribution of the optimal weight $w^{k}$ for each DM and the aggregated optimal weight $w^{\text{agg}}$.

To this end, the joint probability distribution is used, as depicted in Equation (1) below.

(1) $$\begin{array}{c} P\left(w^{\text{agg}},w^{k}|A_{B}^{k},A_{W}^{k}\right) \end{array}$$

Combining Bayes Theorem with Equation (1) provides Equation (2).

(2) $$P\left(w^{\text{agg}},w^{k}|A_{B}^{k},A_{W}^{k}\right)\propto P\left(A_{B}^{k},A_{W}^{k}|w^{\text{agg}},w^{k}\right)P\left(w^{\text{agg}},w^{k}\right)\begin{array}{c} =P\left(w^{\text{agg}}\right)\prod_{1}^{K}P\left(A_{W}^{k}|w^{k}\right)P\left(A_{B}^{k}|w^{k}\right)P\left(w^{k}|w^{\text{agg}}\right) \end{array}$$

To proceed with the computation of the posterior distribution, the variables in Equation (4) are required to be specified. On this basis, the following probabilistic model is established.

(3) $$\begin{array}{c} A_{W}^{k}|w^{k}∼\text{multinomial}\left(w^{k}\right),\forall k=1,\ldots ,K \end{array}$$

(4) $$\begin{array}{c} A_{B}^{k}|w^{k}∼\text{multinomial}\left(1/w^{k}\right),\forall k=1,\ldots ,K \end{array}$$

In the above Equations (3), (4), the multinomial represents $A_{B}^{k}$ and $A_{W}^{k}$ can be modeled by multinomial distribution.

For determining the weight $w^{k}$ in the multinomial distribution, the Dirichlet distribution, as illustrated by Equation (5) below, will serve as the prior distribution to the model $w^{k}$ given its non-negativity and sum-to-one properties.

(5) $$\begin{array}{c} \text{Dir}\left(w|\alpha \right)∼1/B\left(\alpha \right)\prod_{i=1}^{n}w_{j}^{\alpha_{j}‐1},\alpha \in R^{n} \end{array}$$

For $w^{k}$ in Equation (3) or Equation (4), when $w^{\text{agg}}$ is given, it is expected to be in the proximity of $w^{\text{agg}}$. Therefore, the Dirichlet distribution has to be reparametrized, as depicted by Equation (6) below

(6) $$\begin{array}{c} w^{k}|w^{\text{agg}}∼\text{Dir}\left(\gamma \times w^{\text{agg}}\right),\forall k=1,\ldots ,K \end{array}$$

where $w^{\text{agg}}$ denotes the mean of the distribution, and the non-negative parameter γ denotes the closeness between $w^{k}$ and $w^{\text{agg}}$. To model γ, the gamma distribution is adopted, as shown in Equation (7) below

(7) $$\begin{array}{c} \gamma ∼\Gamma \left(a,b\right) \end{array}$$

where a and b are the shape parameters, both set to 0.1.

The model defined above does not yield a closed-form solution. To compute the posterior distribution in Equation (2), Markov Chain Monte Carlo (MCMC) methods are employed, with the “Just Another Gibbs Sampler (JAGS)” utilized for random sample generation [61].

Further, following a sampling procedure encompassing model definition, compilation, initialization, adaptation and burn-in, and monitoring [61], S samples are obtained from the posterior distribution of $w^{\text{agg}}$, which allows for examining the preferences of DMs. These samples are taken first to generate a credal ranking of the determining criteria. This confidence of inter-criteria superiority is another aspect of Bayesian BWM that distinguishes it from the original BWM, which is defined by Equation (8) below

(8) $$\begin{array}{c} P\left(c_{i}>c_{j}\right)=\int I_{\left(w_{i}^{\text{agg}}>w_{j}^{\text{agg}}\right)}P\left(w^{\text{agg}}\right) \end{array}$$

where I equals 1 if the condition in the subscript bears true and 0 otherwise, and $P\left(w^{\text{agg}}\right)$ is the posterior distribution of $w^{\text{agg}}$. Combining the above mentioned that S samples are generated from the posterior distribution of $w^{\text{agg}}$ and thus Equation (8) can be reformulated as Equations (9), (10) below

(9) $$\begin{array}{c} P\left(c_{i}>c_{j}\right)=\frac{1}{S}\sum_{s=1}^{S}I_{\left(w_{i}^{{\text{agg}}_{q}}>w_{j}^{{\text{agg}}_{q}}\right)} \end{array}$$

(10) $$\begin{array}{c} P\left(c_{j}>c_{i}\right)=\frac{1}{S}\sum_{s=1}^{S}I_{\left(w_{j}^{{\text{agg}}_{q}}>w_{i}^{{\text{agg}}_{q}}\right)} \end{array}$$

where $w^{{\text{agg}}_{q}}$ denotes the q th sample of $w^{\text{agg}}$ among the MCMC samples.

### 3.2 HDBSCAN

With the sustainability evaluation framework established for takeaway businesses in the OFD service supply chain, we perform a cluster analysis of the aggregated data. Due to the relatively low dimensionality of the data used, we opt for the density-based clustering method, a popular technique for clustering low-dimensional data [62]. HDBSCAN [63], an enhanced extension of the well-known Density-Based Spatial Clustering of Applications with Noise (DBSCAN), has demonstrated superiority across various real-world datasets [64]. HDBSCAN excels in identifying clusters with varying densities and exhibits robustness in parameter selection [65], that is, it does not require the user to further input arbitrary or biasing parameters except for the minimum cluster size [66]. Leveraging its hierarchical nature, HDBSCAN converts the complex single-linkage hierarchy to a simplified tree of candidate clusters and extracts a flat cluster via local cuts on the tree [67]. The advantages displayed by this algorithm have facilitated it to be widely implemented in diverse research fields such as new algorithm improvements [[67], [68], [69]] and real-world applications like location-based services [70], visualizing correlated motion on protein structures [66]. Also, the HDBSCAN library [65] provides strong support for implementing the algorithm and options for the selection of metric distance. Building on this foundation, we utilize HDBSCAN to conduct the clustering analysis, following these main steps:

*Step 1* Compute the mutual reachability distance

To begin with, some notions need to be clarified. The core points in DBSCAN refer to those objects that have at least *minPts* data points within the ε radius and those in the ε neighborhood of the core points are defined as border points and they are not considered HDBSCAN. Then, the core distance $d_{\text{core}}\left(∙\right)$ of an object $x_{p}\in X$ is the distance from $x_{p}$ to its nearest *minPts* neighbor, where X denotes the data set. Given this, the mutual reachability distance between two objects $x_{p}$ and $x_{q}$, both in X, is computed by the following Equation (11)

(11) $$\begin{array}{c} d_{\text{mreach}}=\max\left\{d_{\text{core}}\left(x_{p}\right),d_{\text{core}}\left(x_{q}\right),d\left(x_{p},x_{q}\right)\right\} \end{array}$$

where $d\left(x_{p},x_{q}\right)$ denotes the normal distance calculated based on the selected distance metric, such as Euclidean distance. Through the core distance, the sparse points will be separated from other points, thus making the clustering more robust to noise.

*Step 2* Build the minimum spanning tree

With the mutual reachable distance, the data set X can be regarded as a complete mutual reachability graph $G_{\text{minPts}}$, in which the data points are the vertices and the connections between any two points are the edges, and the weight of the edge is equal to the mutual reachable distance between these points. Having the mutual reachability graph $G_{\text{minPts}}$, the minimum spanning tree can be built based on an ordinary list search.

*Step 3* Build the cluster hierarchy

In the minimum spanning tree, the extended dendrogram can be constructed by iteratively deleting all edges from the minimum spanning tree in descending order of weights. More specifically, before each deletion, set the scale value of the dendrogram of the current hierarchy to the weight of the edge to be deleted. After each deletion, assign a label to the connected component that contains the end vertex of the deleted edge. For the acquisition of the next hierarchy level: if a component still has at least one edge, a new cluster label is assigned, otherwise, it is assigned a null label, e.g. noise.

*Step 4* Compact the cluster hierarchy

Extracting only significant clusters from the large and complicated cluster hierarchy is a fundamental problem. To this end, a simplification procedure based on continuous-valued probability density functions for HDBSCAN is conducted to condense the complex cluster hierarchy into a smaller tree. If two or more subcomponents of a cluster contain at least *minPts* objects, then the cluster split is viewed as a true split. If they have fewer than *minPts* objects, it is deemed spurious, then the cluster has disappeared at this density level. However, if only one of the subcomponents contains fewer than *minPts* objects, then the cluster shrinks but remains connected.

*Step 5* Extract the stable cluster

Given the condensed cluster hierarchy, one can extract clusters from it. For a finite data set X, the stability of the cluster $C_{i}$ is defined by the following Equation (12)

(12) $$S\left(C_{i}\right)=\sum_{x_{j}\in C_{i}}\left(\lambda_{\max}\left(x_{j},C_{i}\right)‐\lambda_{\min}\left(C_{i}\right)\right)=\sum_{x_{j}\in C_{i}}\left(\frac{1}{\varepsilon_{\min}\left(x_{j},C_{i}\right)}‐\frac{1}{\varepsilon_{\max}\left(C_{i}\right)}\right)$$

where $\lambda_{\max}\left(x_{j},C_{i}\right)$ is the maximum density level beyond which the cluster $C_{i}$ disappears or splits and $\lambda_{\min}\left(C_{i}\right)$ is the minimum density level at which the cluster $C_{i}$ appears. Correspondingly, $\varepsilon_{\max}\left(C_{i}\right)$ and $\varepsilon_{\min}\left(x_{j},C_{i}\right)$ are the values concerning the threshold ε. To obtain the flat cluster, an optimization problem by maximizing the sum of the stabilities of the extracted clusters is formulated, as depicted in the following Equation (13)

(13) $$\begin{array}{c} \max_{\delta_{2},\cdots ,\delta_{k}}J=\sum_{i=2}^{k}\delta_{i}S\left(C_{i}\right)\\ s.t.\;\left\{ \begin{array}{c} \delta_{i}\in \left\{0,1\right\},i=2,\cdots ,k\\ \sum_{j\epsilon I_{h}}\delta_{j}=1,\forall h\epsilon L \end{array}\right. \end{array}$$

where $\delta_{i}$ is a binary variable indicating whether the cluster $C_{i}$ is in the flat solution. $L=\left\{h|C_{h}\;\text{is}\;a\;\text{leaf}\;\text{cluster}\right\}$ is the set of the leaf cluster and $I_{h}$ is the set of all clusters on the paths from $C_{h}$ to the root excluded. For this optimization problem, the selection algorithm of HDBSCAN gives the solution. It traverses every node in the condense tree bottom-up except the root and recursively propagates and updates the total stability of the selected cluster in the subtree to ensure the cluster with the highest stability.

## 4 Case study

In this section, we apply the Bayesian BWM to a real-world case study to derive the weights of the sustainability criteria of the takeaway businesses in the OFD service supply chain in Chongqing, China. Based on the processed data points, we leverage HDBSCAN to categorize them into different clusters, thereby unveiling the sustainability levels of the takeaway businesses.

### 4.1 Criteria determination

Section 2 demonstrates the multifaceted nature of sustainability evaluation for takeaway businesses in the OFD service supply chain, involving various criteria. To enhance the discriminative power of these criteria and mitigate groupthink as much as possible, we integrated perspectives from academia and industry experts, aligning with the findings of Mueller et al. [71]. Three professors from distinct faculties at Chongqing University, specializing in operations research and sustainability, contributed diverse expertise. Additionally, four market managers from the *Ele.me* OFD platform were invited to participate in a structured questionnaire. Furthermore, when soliciting decision opinions from university professors and market managers through questionnaires, we employed online distribution methods to ensure the participation of all individuals, fostering divergent ideas and multi-lateral decision-making. This approach may also alleviate reputational pressures on decision-makers and minimize bias [72,73].

**Fig. 1:**
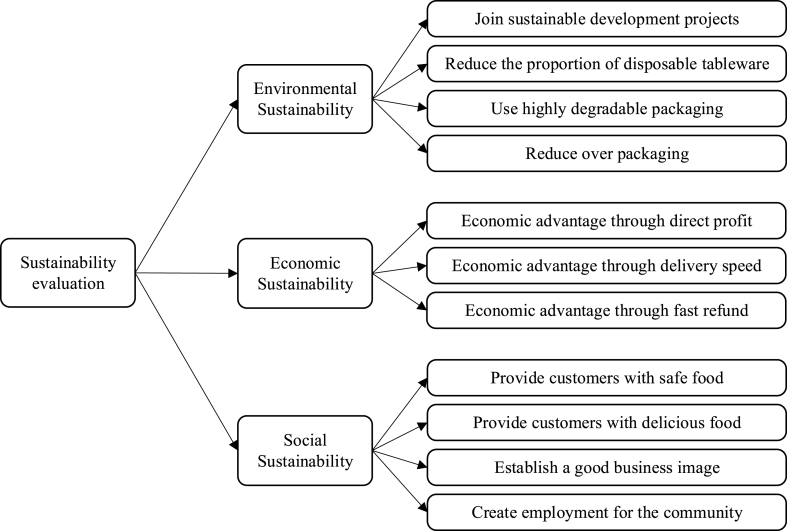
Sustainability evaluation framework for the takeaway businesses.

To facilitate ease of use and efficient screening of evaluation criteria [74], without inducing cognitive overload or excessive burden on invited experts [75], the questionnaire incorporates a 5-point Likert scale. Then, we establish evaluation criteria based on final scores and cutoff values [55]. Recognizing the need for tailored decision criteria [76], discussions with experts delved into sub-criteria like package degradability, food safety, and business image. Drawing on their insights, we finalized a more comprehensive evaluation hierarchy presented in Fig. 1. This involved the addition of criteria aligned with the unique features of takeaway.

### 4.2 Data collection

This paper primarily focuses on the sustainability of takeaway businesses within the context of instant food packaging and delivery, with less emphasis on other aspects like food procurement, storage, preparation, and cooking. Data collection involves two main steps. Initially, we gathered basic data from takeaway businesses on the *Ele.me* OFD platform in Chongqing, China. This selection specifically includes restaurant businesses while excluding others like supermarkets, convenience stores, pharmacies, and fresh food stores. Subsequently, we collect field data based on the defined criteria.

Given the dispersion of businesses across all Chongqing districts in the first step, we concentrate data collection efforts on eight key districts. After considering data availability and relevance, we successfully gather information from 564 takeaway businesses. The Max-Min normalization method is then applied to render the collected data dimensionless.

Simultaneously, in alignment with the Bayesian BWM, we seek scores from experts. Questionnaires are distributed to 12 marketing managers of the *Ele.me* OFD platform, with managers from Shanghai (5), Chongqing (4), and Henan (3). To ensure the managers have adequate information for comparisons and scoring, supplementary documents outlining Bayesian BWM steps and sustainability criteria for takeaway businesses are provided.

### 4.3 Criteria importance

#### 4.3.1 Weights of criteria

Regarding the weightings outlined in Table 1, economic sustainability takes precedence, succeeded by social sustainability, and ultimately, environmental sustainability. For market managers and takeaway businesses, the primary focus is navigating intense market competition and subsequently generating profits [16]. With the expanding emphasis on corporate social responsibility, the integration of “society” and “business” is becoming more economically viable [77]. However, the pursuit of one immediate priority often conflicts with another. Particularly for environmental sustainability, while transformative, it's essential to recognize that this shift will not occur instantaneously; it might take 2–5 years or even up to 10 years to witness tangible results [18].

**Table 1:** Weights of main criteria and sub-criteria.

Main criteria	Weight	Sub-criteria	Local weight	Global weight
Environmental (C_1_)	0.1556	Join sustainable development projects (C_11_)	0.0907	0.0302
		Reduce the proportion of disposable tableware (C_12_)	0.2679	0.0893
		Use highly degradable packaging (C_13_)	0.4906	0.1635
		Reduce over packaging (C_14_)	0.1508	0.0503
Economic (C_2_)	0.4919	Economic advantage through direct profit (C_21_)	0.4770	0.1590
		Economic advantage through delivery speed (C_22_)	0.3944	0.1315
		Economic advantage through fast refund (C_23_)	0.1286	0.0429
Social (C_3_)	0.3525	Provide customers with safe food (C_31_)	0.4924	0.1641
		Provide customers with delicious food (C_32_)	0.2142	0.0714
		Establish a good business image (C_33_)	0.1924	0.0641
		Create employment for the community (C_34_)	0.1011	0.0337

Examining the global weights, the most prominent sub-criteria include “Provide customers with safe food,” “Use highly degradable packaging,” “Economic advantage through direct profit,” and “Economic advantage through delivery speed,” with weights surpassing 0.13. Notably, food safety holds the utmost importance, intricately linked to the nature of the restaurant industry. Despite being a constant challenge, especially for those transitioning to green operations, prioritizing food safety remains crucial [18]. Following closely is the use of highly degradable packaging. While takeaway services offer convenience, the subsequent accumulation of packaging waste demands attention. Opting for environmentally safe and biodegradable packaging materials and tableware becomes imperative [40]. Fortunately, certain OFD platforms, like *Ele.me* in China, are actively promoting environmental sustainability by endorsing biodegradable packaging and tableware, implementing tableware recycling and remanufacturing in pilot cities, and supporting eco-friendly businesses [51].

Subsequently, the profitability of takeaway businesses ranks third. Managers bear the responsibility of ensuring business survival and maximizing profits. By maintaining or increasing profitability, the needs of environmental and social sustainability can be better addressed [18]. The fourth priority is delivery speed. The essence of takeaway lies in providing customers with convenient services, heavily dependent on swift delivery. Given that many food items are unsuitable for prolonged storage, and electronic food businesses are time-sensitive, delivery speed becomes a crucial factor [11].

#### 4.3.2 Credal ranking

In discerning the relative importance among criteria, the Bayesian BWM inherently captures decision-makers’ preferences in a probabilistic manner.

**Fig. 2:**
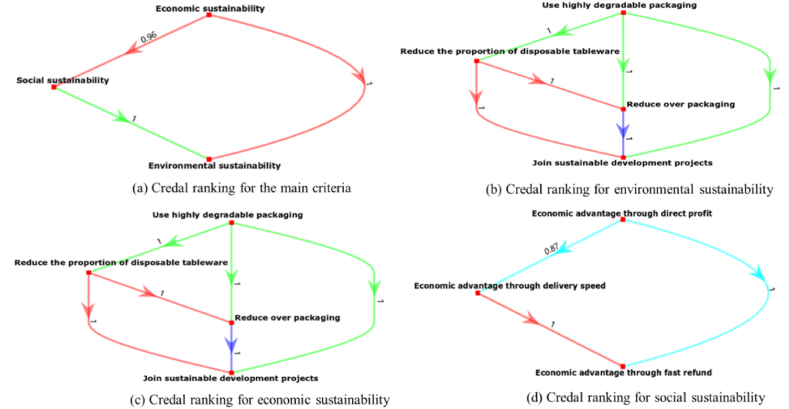
Credal ranking for takeaway businesses' sustainability.

Fig. 2(a) illustrates the prevailing importance of economic sustainability over the other two criteria. From the perspective of OFD platform managers, the profitability of takeaway businesses remains paramount. While the ultimate goal may be sustainable development, businesses are inherently driven by financial considerations. Aligning positive environmental outcomes with financial success does support green operation initiatives [18]. Social sustainability holds the second position and remains significant compared to environmental sustainability, with a confidence level of 1. However, the probability value of 0.96 between economic and social sustainability indicates that some managers perceive social sustainability as more crucial. Businesses increasingly value fulfilling social responsibilities to diverse stakeholders, fostering community connections, and promoting synergies among the economy, environment, and society for long-term business value growth [31]. Environmental sustainability ranks the lowest in priority, aligning with the observation that “none of the restaurateurs mentioned sustainability as being important” [33]. In the short term, businesses and managers tend to prioritize profit, but sustainable strategies can contribute to cost reduction in the long run [34].

Regarding environmental sustainability in Fig. 2(b), “Use highly degradable packaging” takes precedence among the four criteria. The utilization of biodegradable packaging materials in takeaway food exemplifies the incorporation of sustainability into online food ordering [78]. Currently, completely banning disposable products is challenging; hence, gradually reducing the use of disposable tableware in each order and exploring environmentally friendly tableware materials could be a viable solution. The probability value of 1 for the criterion “Reduce the proportion of disposable tableware” indicates its significance compared to “Reducing over packaging” and “Join sustainable development projects.” To mitigate the negative impact of packaging waste, tableware waste, and food residue waste, reducing the use of disposable tableware is imperative. While businesses may need to use some plastic products for hygiene and convenience, they must exert better control, minimize usage, and substitute them with sustainable alternatives when feasible [46]. “Reducing over packaging” holds greater importance than “Join sustainable development projects,” suggesting high value placed by managers. Less packaging translates to less waste. While reducing over packaging positively affects the environmental friendliness of the product, it might compromise perceived quality and convenience [42]. For the criterion “Join sustainable development projects,” the other three criteria all hold significant positions, each with a probability value of 1.

Concerning economic sustainability in Fig. 2(c), the criterion “Economic advantage through direct profit” unequivocally holds the highest priority with a value of 1. This prioritization stems from the understanding that for any business to thrive in market competition, the financial outcomes must be profitable to align with expectations [34]. Only through profitable financial outcomes can subsequent sustainable development initiatives be supported effectively [18]. A probability value of 1 indicates that some managers also highly value profitability achieved through delivery speed. Considering the nature of OFD, delivery speed significantly impacts customer waiting time [11]. Providing fast and reliable delivery services not only meets customer expectations but also contributes to profitability. “Economic advantage through fast refund” stands as the least crucial criterion. Nevertheless, supporting a fast refund is a typical service remedy employed by businesses to enhance customer satisfaction and foster repeated purchase intentions [50], thereby indirectly contributing to economic advantage.

Social sustainability is embodied in a company's policies that safeguard and uphold the health and safety of customers. Therefore, for takeaway businesses, ensuring the provision of safe food stands as a top priority, supported by the probability value of 1, as illustrated in Fig. 2(d). The criterion of “Providing customers with delicious food” surpasses only food safety and holds more significance than “Establish a good corporate image,” with a confidence level of 0.73. This alignment is closely tied to our case study focus on catering takeaway businesses. The quality of food, encompassing packaging, taste, temperature, and freshness, constitutes vital considerations for customers when placing an order [11]. The criterion “Establish a good corporate image” still maintains higher priority than “Create employment for the community,” as customers are more inclined to patronize reputable businesses. For the criterion of “Create employment for the community,” the probability value of 1 indicates that managers do not view it as crucial as the other three, despite takeaway businesses significantly contributing to community employment. In China, for instance, as of 2019, the OFD industry employed approximately 13 million delivery personnel, constituting 1% of the country's population [79].

### 4.4 Clustering analysis

Leveraging the data collected for each criterion along with the criterion weights, we employ the HDBSCAN method to unveil clusters of takeaway businesses contingent on their sustainability level.

**Fig. 3:**
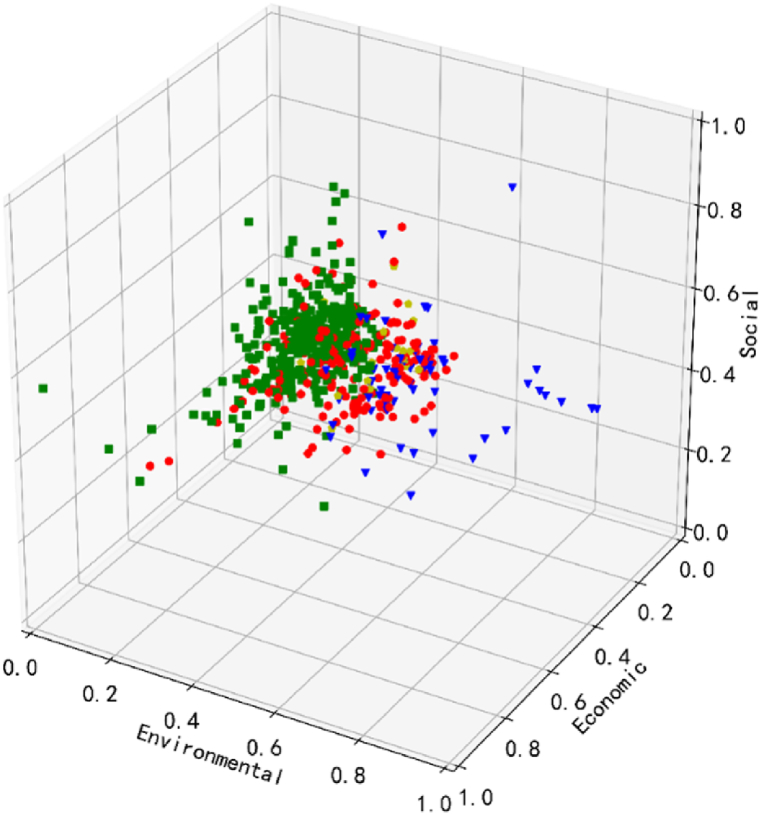
Clustering distribution.

Following the aforementioned steps, the data points are ultimately grouped into four distinct categories, as illustrated in Fig. 3 below.

In Fig. 3, the most prominent category is characterized by the green rectangle, indicating the highest number of takeaway businesses compared to the other three types. Conversely, the blue triangle represents the smallest number of takeaway businesses, most of which are positioned distantly from the green ones. Positioned in between are the other two types of businesses, symbolized by the red circle and the brown pentagon. The close distribution of these two types of businesses suggests a certain similarity between them, yet differences in quantity and the level of environmental sustainability are still discernible.

**Fig. 4:**
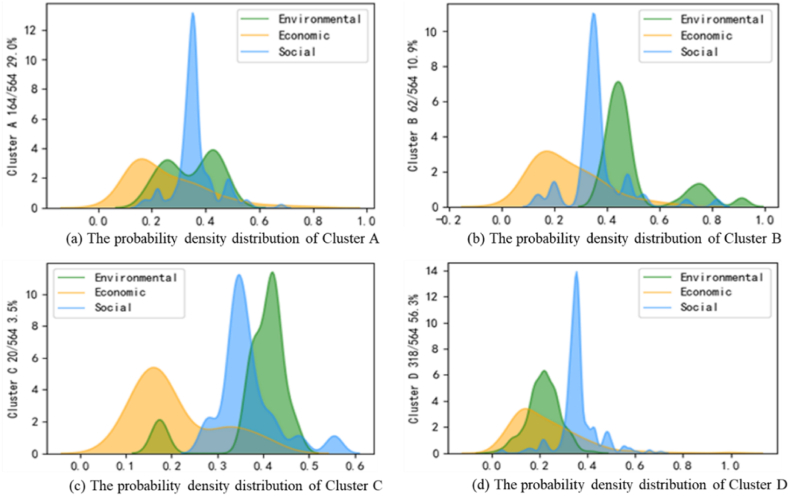
Probability density distribution.

Additionally, we present the probability density distributions of different types of takeaway businesses in Fig. 4 below.

Combining the information presented in Fig. 4, we can summarize the characteristics of takeaway businesses categorized into four clusters:

Cluster A (Low-medium sustainability): Environmental sustainability scores vary between 0.25 and 0.45, featuring a noticeable peak and slight disparities in peaks. Economic sustainability scores hover around 0.18. In comparison, social sustainability scores are higher, primarily around 0.36, with two minor peaks at 0.2 and 0.47, though with minimal business density around these peaks. Cluster A represents 29% of the total sample, ranking second only to Cluster D. While social sustainability is robust, economic and environmental sustainability are moderate.

Cluster B (Medium-high sustainability): For this cluster, environmental sustainability scores surpass Cluster A, mainly ranging between 0.45 and 0.5, with a more concentrated density at the peak. This dimension exhibits three peaks, the latter two centered around 0.75 and 0.9, albeit with minimal distribution density. Economic sustainability scores show marginal differences from Cluster A, fluctuating mainly around 0.19, with improved performance on the right side of the peak. Social sustainability scores remain high, around 0.35, with slight increases evidenced by peaks at 0.7 and 0.82. Cluster B represents 10.9% of the total sample.

Cluster C: (High sustainability): This cluster displays two peaks in environmental sustainability scores. The first is around 0.17, with a sparse distribution, while the second peaks at 0.43, exhibiting a denser distribution. Social sustainability scores mostly fluctuate around 0.36, with a denser distribution. Economic sustainability lags, scoring around 0.15, with a higher density on the right side of the peak. Cluster C accounts for only 3.5% of the total sample. These businesses require economic sustainability enhancement, prompting the OFD platform to consider subsidies or marketing strategies.

Cluster D: (Low sustainability): The businesses in this cluster fare poorly across all sustainability dimensions. Environmental sustainability scores hover around 0.21, indicating worse performance. Social sustainability shows small fluctuations, with a narrower density distribution compared to the other clusters. Economic sustainability is stable but unsatisfactory, lacking a long-tail distribution. Cluster D constitutes 56.3% of the total sample, necessitating attention from the OFD platform managers.

Across the four clusters, differences and similarities emerge, particularly in social and economic sustainability. Concerning social sustainability, business density mainly fluctuates between 0.2 and 0.3, except for Cluster C, which fluctuates between 0.3 and 0.45. Despite minor discrepancies, the density distribution peak is high, indicating better performance in social sustainability. Takeaway businesses in all clusters prioritize food safety, deliciousness, brand image, and employment. Economic sustainability is consistently low, concentrated in the range of 0.1–0.3, highlighting the prevalent lack of economic sustainability among takeaway businesses in China, as reflected in our limited sample data.

## 5 Discussions

In this section, we further discuss the relationship between the sustainability level of takeaway businesses and the cuisine category, as well as their respective business sizes.

### 5.1 Cuisine categories

**Fig. 5:**
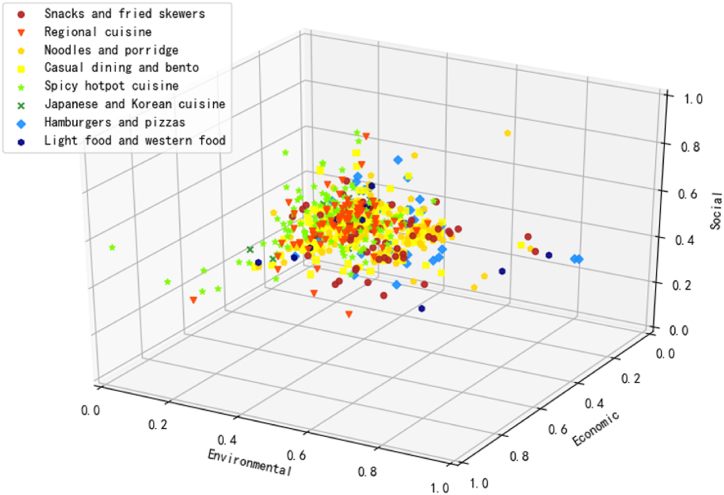
Distribution of cuisine categories.

**Fig. 6:**
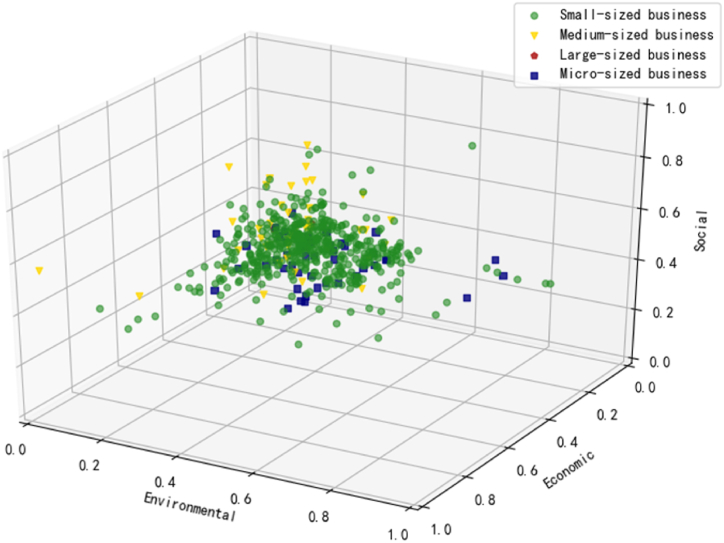
Distribution of business size.

In the preceding subsection, businesses are clustered based on inherent similarities in sustainability performance, treating them as a unified type. To illustrate the distribution of diverse businesses concerning cuisine classification and business size, we present spatial distributions in Fig. 5, Fig. 6 below, respectively.

Illustrated in Fig. 5, the representation of points across the three categories—Japanese and Korean cuisine, hamburgers and pizzas, and Light food and Western food—is relatively modest. Notably, hamburgers and pizzas exhibit the highest overall sustainability performance among all categories. Despite sporadic distributions of other cuisine categories in areas with higher sustainability scores, hamburgers and pizzas stand out. Casual dining and bento, spicy hotpot cuisine, noodles and porridge, regional cuisine, and snacks and fried skewers collectively dominate the cuisine categories, accounting for 26.95%, 21.45%, 14.89%, 14.18%, and 9.57%, respectively.

Furthermore, the 3D spatial aggregation of these cuisines suggests that they tend to be less environmentally and economically sustainable but slightly more socially sustainable. Casual dining and bento, spicy hotpot cuisine, and regional cuisine cluster notably close to the origin. In terms of environmental sustainability, the clustering aligns closely with the city where the case study was conducted. Notably, Chongqing cuisine, particularly hotpot and local dishes, tends to involve more disposable tableware and poses challenges for recycling due to grease contamination. This observation aligns with data from the *Ele.me* OFD platform, indicating that the Chongqing and Sichuan cuisine types significantly impact the volume of tableware used [80].

### 5.2 Business size

Concerning the size distribution, Fig. 6 reveals a concentrated density of small-sized businesses denoted by the green circles, followed by micro and medium-sized establishments, with very few large ones. According to the raw data, these categories account for 83.33%, 8.33%, 7.98%, and 0.35%, respectively. This observation aligns with reports indicating that catering businesses in China predominantly fall into the small and medium-sized categories, characterized by low mechanization and centralization, reflecting a pattern of large quantity and small scale [12].

Fig. 6 clearly indicates that the majority of micro, small, and medium-sized businesses exhibit subpar performance in terms of economic and environmental sustainability while demonstrating relatively better results in social sustainability. For these businesses, economic sustainability often takes precedence, given the imperative to thrive in a fiercely competitive market. Additionally, even if they achieve profitability, the implementation of sustainability plans might be met with reluctance. A notable example is the adoption of biodegradable tableware, which typically incurs higher costs than regular plastic alternatives, subsequently passed on to the customer. This, in turn, may deter customers from choosing the takeaway option, as many are unwilling to bear the additional costs associated with environmentally friendly measures [29,52]. Hence, it becomes crucial for OFD platform managers to comprehensively consider the size and financial capacity of businesses when assisting in formulating sustainability strategies.

## 6 Conclusions

### 6.1 Summary of the research

This paper presents a two-stage methodology to mine the sustainability of takeaway businesses in the OFD service supply chain. Firstly, a case study is conducted in the main district of Chongqing, China, utilizing Bayesian BWM to determine sustainability criteria weights and scores. Economic sustainability is found to be most significant, followed by social and environmental sustainability. Secondly, HDBSCAN is employed to cluster takeaway businesses based on sustainability criteria, revealing four distinct sustainability levels, with a majority demonstrating low sustainability. Despite economic sustainability being prioritized, environmental sustainability is highlighted as a critical concern, with takeaway businesses generally excelling in social sustainability. Furthermore, analysis of sustainability clustering across cuisine categories identifies inadequacies in economic, environmental, and social sustainability within certain food genres, which are linked to specific cities in the study. Examination of sustainability clustering by business size reveals that micro, small, and medium-sized businesses dominate the OFD service supply chain, exhibiting lower environmental and economic sustainability but strong social sustainability performance.

### 6.2 Key implications

#### 6.2.1 Theoretical implications

(1) Enhanced understanding of sustainability priorities

The findings contribute to theoretical advancements by revealing the relative importance of economic, social, and environmental sustainability within the context of takeaway businesses in the OFD service supply chain. This understanding helps in refining existing sustainability frameworks and theories.

(2) Insights into sustainability clustering

The utilization of HDBSCAN for clustering takeaway businesses based on sustainability criteria offers insights into the nuanced levels of sustainability within the OFD sector. This contributes to theoretical understanding by providing a methodological framework for assessing and categorizing sustainability performance.

(3) Intersection of sustainability and business size

By exploring the relationship between sustainability clustering and business size, the study sheds light on the dynamics between organizational characteristics and sustainability outcomes. This provides theoretical insights into how different business sizes may prioritize and manage sustainability initiatives.

#### 6.2.2 *b.* Practical implications

(1) Fostering collaboration and forging partnerships

The identification of sustainability inadequacies within takeaway businesses presents an opportune moment for fostering collaboration and forging partnerships within the OFD service supply chain. Beyond merely identifying deficiencies, this revelation prompts OFD platform administrators, takeaway businesses, suppliers, and various stakeholders to engage in collective action aimed at rectifying sustainability shortcomings. By pooling their resources and expertise, these stakeholders can collaboratively develop and implement sustainable sourcing practices. This entails ensuring the availability of environmentally friendly ingredients and materials, thereby mitigating the ecological footprint associated with OFD operations.

The feasibility of fostering collaboration and forging partnerships within the OFD service supply chain is evident, yet it requires overcoming the influence of OFD platform dominance on the willingness of other chain members to cooperate. Therefore, practicality primarily lies in the collective commitment of stakeholders to address sustainability inadequacies.

(2) Prioritizing economic sustainability

Platform managers and takeaway businesses prioritize economic sustainability. Therefore, ensuring continuous profitability is essential for maintaining competitiveness in a competitive market. Takeaway businesses, especially micro, small, and medium-sized ones, are encouraged to leverage data and information from OFD platforms to expand marketing channels, formulate diverse strategies, and reduce costs. OFD platforms can support takeaway businesses by offering monetary or non-monetary subsidies and lowering commission rates, thereby maintaining existing partnerships and attracting new ones. Additionally, takeaway businesses should focus on providing high-quality food, improving the efficiency of food preparation, and enhancing after-sales service to attract and retain customers.

Prioritizing economic sustainability is feasible for takeaway businesses and OFD platforms because survival is paramount in the current fiercely competitive environment. However, in the execution process, a dilemma arises concerning the high commissions charged by OFD platforms. This issue necessitates thorough negotiations between takeaway businesses and OFD platforms, and if necessary, joint bargaining with multiple enterprises against the platform.

(3) Enhancing environmental sustainability

While economic sustainability takes precedence in the OFD sector, addressing environmental concerns is equally imperative. Many takeaway businesses struggle with low environmental sustainability, necessitating proactive measures to mitigate their ecological footprint. Transitioning to eco-friendly alternatives in packaging and tableware, along with reducing resource and energy consumption, is crucial. Additionally, incentivizing biodegradable packaging adoption and minimizing food waste through optimized inventory management and donations are essential. Adopting energy-efficient practices, such as using electric vehicles and implementing energy-saving measures, further enhances environmental sustainability. OFD platforms play a key role in guiding businesses towards sustainability by supporting eco-friendly initiatives and investing in research and development on environmentally-friendly products. Collaboration among stakeholders is vital to drive meaningful change and foster environmental responsibility within the OFD sector.

The recommendation to enhance environmental sustainability is feasible, especially now that the *Ele.me* OFD platform has begun promoting biodegradable packaging. However, it is important to note that implementing environmental sustainability initiatives among takeaway businesses requires time and investment, particularly for small and medium-sized businesses. Therefore, government and platform support through environmental subsidies becomes crucial.

(4) Promoting social sustainability

Promoting social sustainability in the OFD service supply chain involves fulfilling various responsibilities to stakeholders, including providing safe food, employing delivery personnel to enhance community vitality, and ensuring equitable treatment for employees. While most takeaway businesses demonstrate strong performance, some require platform oversight, such as monitoring food safety and implementing rewards and penalties. Leveraging data mining techniques, platforms can assess the social sustainability of takeaway businesses, with top performers receiving enhanced exposure and marketing. This approach indirectly supports economic sustainability, fostering exemplary businesses for emulation.

Promoting social sustainability is also a feasible recommendation. Ensuring food safety and enhancing community employment are among the initiatives currently being implemented by takeaway businesses. Additionally, for OFD platforms, using data mining techniques to monitor and improve the social sustainability of takeaway businesses poses certain challenges, as it may require additional allocation of resources such as manpower and equipment.

(5) Adapting to regulatory changes

Armed with evidence-based findings, policymakers are empowered to design and enact targeted policies and regulations aimed at fostering sustainability within the OFD sector. However, regulatory changes significantly influence takeaway businesses’ adoption of sustainable practices. For instance, mandates to reduce single-use plastics or embrace eco-friendly packaging necessitate adaptation. While initially posing financial burdens, these regulations offer avenues for environmental stewardship and enhancing public perception. Moreover, regulatory pressures spur innovation, leading to investments in renewable energy, waste reduction, and eco-friendly delivery methods in the OFD sector. However, smaller takeaway businesses face challenges in meeting stringent standards due to resource constraints, impacting profitability and competitiveness. Strategic collaborations with policymakers, industry associations, and consumer demand for eco-conscious options offer pathways for takeaway businesses to develop tailored sustainability strategies.

Adapting to regulatory policy changes is feasible for both takeaway businesses and OFD platforms. However, these changes often bring various challenges initially, aligning with real-world scenarios. Despite challenges, regulatory changes drive sustainability transformation, fostering innovation and collaboration in the OFD sector. Therefore, the recommendation's practicality depends on their ability to navigate challenges and seize opportunities.

### 6.3 Theoretical and practical contributions

#### 6.3.1 Theoretical contributions

The sustainability of takeaway businesses is examined and a generic MCDM framework for evaluating the sustainability of takeaway businesses is proposed. This framework provides a systematic and comprehensive method for evaluating sustainability criteria and clustering businesses based on their sustainability performance. Besides, the introduction of a two-stage approach integrating Bayesian BWM and HDBSCAN contributes to the theoretical advancement of sustainability assessment methodologies within the context of takeaway businesses in the OFD service supply chain.

#### 6.3.2 Practical contributions

The proposed MCDM framework is applied to a real-world case study that has a significant contribution to sustainability practice in takeaway businesses. The identification of economic sustainability as paramount and the recognition of emerging environmental concerns provide practical guidance for OFD platform administrators and takeaway businesses in crafting sustainability strategies. This entails resource allocation and targeted initiatives to bolster economic viability and address environmental challenges. Additionally, clustering takeaway businesses into distinct sustainability levels enables tailored intervention strategies, enhancing sustainability performance and addressing sector-specific challenges. The observed disparity in sustainability performance among micro, small, and medium-sized businesses underscores the need for targeted support initiatives, such as capacity-building programs and resource access, to bolster sustainability practices. Furthermore, insights regarding sustainability clustering and its relationship with business size inform policymakers and regulatory authorities, facilitating the development of policies aimed at fostering sustainability practices within the OFD service supply chain.

### 6.4 Limitations and future research intentions

#### 6.4.1 Limitations

In addition to the aforementioned findings, we acknowledge that every study may encounter limitations, and ours is no exception. Our investigation primarily delves into the sustainability of takeaway businesses within the OFD service supply chain, with a specific focus on Chongqing as our research case. Consequently, our study's geographical scope is confined to a particular region in China, rather than undertaking a multi-regional exploration of the sustainability of takeaway businesses. Furthermore, the focused nature of our research subject has resulted in a limited number of OFD service providers represented in the study, potentially leading to a narrower examination of the OFD service.

#### 6.4.2 Future research intentions

Drawing upon the limitations of our study, future research endeavors could entail enlarging the sample size and conducting comparative assessments of sustainability levels among takeaway businesses across varied regions in China. Moreover, we suggest an examination of sustainability considerations pertinent to takeaway packaging and delivery. Subsequent research efforts may involve the ongoing surveillance and appraisal of sustainability performance throughout the entirety of the supply chain, encompassing activities from food procurement and storage to preparation and production stages.

## Institutional ethics committee provided approval

The study was approved by the Academic Ethics Committee of Chongqing University of Science and Technology (reference number 2023LLSC0237).

## Data availability statement

The authors do not have permission to share data.

## CRediT authorship contribution statement

**Longxiao Li:** Writing – original draft, Methodology, Funding acquisition, Formal analysis, Conceptualization. **Zusheng Zhang:** Writing – original draft, Validation, Software, Methodology, Data curation. **Xixi Li:** Resources. **Jiafu Su:** Writing – review & editing, Supervision, Resources, Project administration, Methodology. **Yanling Jiang:** Writing – review & editing, Resources. **Jun Cao:** Resources, Funding acquisition, Data curation. **Fangsu Zhao:** Resources, Funding acquisition, Formal analysis.

## Declaration of competing interest

The authors declare the following financial interests/personal relationships which may be considered as potential competing interests:Longxiao Li, Jun Cao and Fangsu Zhao reports financial support was provided by 10.13039/501100007957Chongqing Municipal Education Commission. If there are other authors, they declare that they have no known competing financial interests or personal relationships that could have appeared to influence the work reported in this paper.
