# Supplementary material for: Mining the sustainability of takeaway businesses in online food delivery service supply chain
Source: Heliyon. 2024 Mar 10;10(6):e27938. doi: 10.1016/j.heliyon.2024.e27938 (PMC10950709; doi:10.1016/j.heliyon.2024.e27938)
Supplement: Multimedia component 1 [file mmc1.docx]

**Questionnaire 1**

Dear esteemed expert,

I hope this letter finds you well. Our team is conducting a questionnaire on the topic “Sustainability evaluation of takeaway businesses in online food delivery service supply chain”. We sincerely appreciate your participation. The purpose of this questionnaire is to determine the sustainability evaluation criteria for takeaway businesses. Therefore, we kindly request your thoughtful consideration in assigning importance scores to each criterion presented in Tables 2-4, utilizing the 5-point Likert scale outlined in Table 1.

Your insights are immensely valuable, and we appreciate your time and effort in contributing to our research.

Best regards,

Longxiao Li

**Table 1** The 5-point Likert scale

| **Values** | **Meanings** |
| --- | --- |
| 1 | Equally important |
| 2 | Between equally important and important |
| 3 | Important |
| 4 | Between important and very important |
| 5 | Very important |

**Table 2** The criteria of environmental sustainability

| Criteria | Descriptions | Score (1-5) |
| --- | --- | --- |
| Implement sustainable food and menu practices | Support local, seasonal products, introduce organic, ecological, and healthy products, and list the food ingredients on the menu. |  |
| Reduce food waste | Implement food recycling and donation programs, while also initiate food waste audits and tracking to reduce the amount of food waste. |  |
| Reduce the proportion of disposable tableware | Avoid using disposable products such as plastic spoons, bowls, and chopsticks, instead of using recyclable paper products or electronic menus. |  |
| Use highly degradable packaging | Use packaging and tableware that is environmentally friendly and biodegradable in response to the demand for sustainability. |  |
| Reduce over packaging | Reduce excessive packaging or unnecessary packaging of food to reduce waste of packaging materials in food service operations. |  |
| Join sustainable development projects | Green Restaurant, Green Seal, Energy Star Pro-gram, ConSERVE Solutions for Sustainability, Sustainable Foodservice, TFC Recycling. |  |
| Reduce the resource and energy use and green-house gas emission | Use recycled natural resources, implement energy audits and management programs, and adopt energy-saving equipment and low-energy consumption design techniques to abate resource and energy consumption and greenhouse gases. |  |

**Table 3** The criteria of economic sustainability

| Criteria | Descriptions | Score (1-5) |
| --- | --- | --- |
| Economic advantage through direct profit | Manage losses and surpluses on food service operations to maximize attainable profits. |  |
| Economic advantage through creating market opportunities | Place great emphasis on the market and create marketing advantages and more opportunities for local restaurant businesses, supply chain partners, and other relevant entities. |  |
| Economic advantage through simplifying product procurement procedures | Reduce the complexity of the ordering process and use single-source purchasing, thereby saving staff time and money. |  |
| Economic advantage through delivery speed | Provide customers with a faster and more convenient online food delivery service that reduces customer waiting time, thereby increasing customer patronage and creating an economic advantage. |  |
| Economic advantage through the fast refund | Provide refund or exchange service to increase customer satisfaction, and purchase frequency, and further increase customer loyalty and the business’s sales and profitability. |  |

**Table 4** The criteria of social sustainability

| Criteria | Descriptions | Score (1-5) |
| --- | --- | --- |
| Provide the customer with safe food | Providing safe and healthy food to customers is the highest priority for food retailers especially those that are going green. |  |
| Provide the customer with delicious food | Serve tasty food to customers where taste, the freshness of ingredients, the temperature of food, and packaging are key considerations, especially in the takeaway delivery scenario. |  |
| Create employment for the local community | Promote member connections, community participation, and prosperity with the community-embedded business via job creation, creative food, and other activities. |  |
| Promote local economic development through procurement practices | Purchase responsibly grown, local, and seasonal products to help the local economy while achieving potential cost-saving and alleviating the impact on the environment. |  |
| Establish a good business image in the local community | Understand customer needs and value customer satisfaction, emphasize the contribution of the restaurant’s operation to the multiple entities in the local community, thus establishing a good business image and serving as a model business that others can emulate. |  |

**Questionnaire 2**

Dear esteemed expert,

I hope this letter finds you well. Our team is conducting a questionnaire on the topic “Sustainability evaluation of takeaway businesses in online food delivery service supply chain”. We sincerely appreciate your participation. This questionnaire aims to explore the relative importance of sustainability evaluation criteria for takeaway businesses in online food delivery service supply chain.

Table 1 below introduces the sustainability evaluation framework for takeaway businesses. It includes 3 main criteria with their respective descriptions and meanings, as well as 11 sub-criteria. In our study, the Bayesian best-worst method is employed to determine the relative importance of the criteria. Therefore, in the following text, we further explain the steps and criteria for using this method. Please refer to Table 2 for details. Tables 3 to 10 present the corresponding expert scoring sheets. Consequently, we kindly ask you to take some time to score the importance of each main criterion in comparison with others, as well as the relative importance of sub-criteria.

Once again, we express our sincere appreciation for your support in our research endeavors. Wishing you the very best.

Best regards,

Longxiao Li

**Table 1** Sustainability evaluation framework for takeaway businesses

| **Goal** | **Main criteria** | **Meanings** | **Sub-criteria** |
| --- | --- | --- | --- |
| Sustainability evaluation of takeaway businesses in online food delivery service supply chain | Environmental  sustainability | The takeaway businesses demonstrate environmentally responsible practices by incorporating on-demand delivery services. | Join sustainable development projects |
|  |  |  | Reduce the proportion of disposable tableware |
|  |  |  | Use highly degradable packaging |
|  |  |  | Reduce over packaging |
|  | Economic  sustainability | The takeaway businesses exhibit both direct profitability and indirect economic sustainability. | Economic advantage through direct profit |
|  |  |  | Economic advantage through delivery speed |
|  |  |  | Economic advantage through fast refund |
|  | Social  sustainability | The takeaway businesses demonstrate their commitment to corporate social responsibility for various stakeholders, including customers, delivery personnel, and local communities. | Provide customers with safe food |
|  |  |  | Provide customers with delicious food |
|  |  |  | Establish a good business image |
|  |  |  | Create employment for the community |

**The Bayesian best-worst method: scoring steps**

Step 1: Please identify the best (most important) and worst (least important) criteria among all the criteria.

Step 2: Please determine the importance of the best criteria relative to other criteria, using a numerical scale of 1-9, with the meanings represented as shown in Table 2.

Step 3: Please determine the importance of other criteria relative to the worst criteria, using a numerical scale of 1-9.

**Table 2** The meanings of numerical values of Bayesian best-worst method

| **Values** | **Meanings** |
| --- | --- |
| 1 | Equally important |
| 2 | Between equally important and slightly important |
| 3 | Slightly important |
| 4 | Between slightly important and important |
| 5 | Important |
| 6 | Between important and very important |
| 7 | Very important |
| 8 | Between very important and absolutely important |
| 9 | Absolutely important |

**Table 3** The comparison between the most important criteria with other criteria

(Main criteria)

| Main criteria | Environmental  sustainability | Economic  sustainability | Social  sustainability |
| --- | --- | --- | --- |
| The most important criteria |  |  |  |

**Table 4** The comparison between the other criteria relative with the least important criteria (Main criteria)

| Main criteria | The least important criteria |
| --- | --- |
| Environmental sustainability |  |
| Economic sustainability  Social sustainability |  |

**Table 5** The comparison between the most important criteria with other criteria

(Sub-criteria of Environmental sustainability)

| Sub-criteria | Join sustainable development projects | Reduce the proportion of disposable tableware | Use highly degradable packaging | Reduce over packaging |
| --- | --- | --- | --- | --- |
| The most important criteria |  |  |  |  |

**Table 6** The comparison between the other criteria relative with the least important criteria (Sub-criteria of Environmental sustainability)

| Sub-criteria | The least important criteria |
| --- | --- |
| Join sustainable development projects |  |
| Reduce the proportion of disposable tableware  Use highly degradable packaging  Reduce over packaging |  |

**Table 7** The comparison between the most important criteria with other criteria

(Sub-criteria of Economic sustainability)

| Sub-criteria | Economic advantage through direct profit | Economic advantage through delivery speed | Economic advantage through fast refund |
| --- | --- | --- | --- |
| The most important criteria |  |  |  |

**Table 8** The comparison between the other criteria relative with the least important criteria (Sub-criteria of Economic sustainability)

| Sub-criteria | The least important criteria |
| --- | --- |
| Economic advantage through direct profit |  |
| Economic advantage through delivery speed  Economic advantage through fast refund |  |

**Table 9** The comparison between the most important criteria with other criteria

(Sub-criteria of Social sustainability)

| Sub-criteria | Provide customers with safe food | Provide customers with delicious food | Establish a good business image | Create employment for the community |
| --- | --- | --- | --- | --- |
| The most important criteria |  |  |  |  |

**Table 10** The comparison between the other criteria relative with the least important criteria (Sub-criteria of Social sustainability)

| Sub-criteria | The least important criteria |
| --- | --- |
| Provide customers with safe food |  |
| Provide customers with delicious food  Establish a good business image  Create employment for the community |  |
